# Evaluation of a Novel Patient-Centered Methadone Restart Protocol

**DOI:** 10.1001/jamanetworkopen.2025.29393

**Published:** 2025-08-28

**Authors:** Paul J. Christine, Joshua Blum, Alexandra R. Tillman, Hannan M. Braun, Martin Hinrichs, Hermione Hurley, Alia Al-Tayyib

**Affiliations:** 1 Department of General Internal Medicine, Denver Health and Hospital Authority, Denver, Colorado; 2 Division of General Internal Medicine, Department of Medicine, University of Colorado School of Medicine, Aurora; 3 Center for Addiction Medicine, Denver Health and Hospital Authority, Denver, Colorado; 4 Outpatient Behavioral Health Services, Denver Health and Hospital Authority, Denver, Colorado; 5 Division of Infectious Diseases, Department of Medicine, Denver Health and Hospital Authority, Denver, Colorado; 6 Public Health Institute at Denver Health, Denver Health and Hospital Authority, Denver, Colorado; 7 Center for Health Systems Research, Office of Research, Denver Health and Hospital Authority, Denver, Colorado

## Abstract

**Importance:**

In opioid treatment programs (OTPs), restarting methadone after missed doses is common. Current guidelines recommend significant dose decreases but do not account for maintained tolerance.

**Objective:**

To evaluate the safety and treatment retention associated with a novel methadone restart protocol.

**Design, Setting, and Participants:**

This cohort study included participants in a public, safety-net OTP in Colorado, comparing cohorts before (2021) and after (2023) a 2022 protocol change. Eligible participants were patients who restarted methadone after 4 or more missed dosing days. Data were analyzed August 2024 through June 2025.

**Exposure:**

Restart protocol incorporating patient-reported nonprescribed opioid use and individualized assessments to determine restart doses.

**Main Outcomes and Measures:**

Protocol implementation was measured by change in dose before vs after restart, comparing preimplementation and postimplementation periods. Primary outcome was patient safety, measured by emergency department (ED) visits within 7 days of restart (all-cause, opioid withdrawal, and overdose-related) and 7-day and 90-day all-cause mortality. The secondary outcome was 90-day treatment retention. Generalized estimating equations were used to calculate adjusted risk ratios (aRRs) comparing preimplementation and postimplementation periods, controlling for demographics and comorbid substance use. Patient and clinician satisfaction and perceived safety were surveyed.

**Results:**

A total of 786 patients were included in the preimplementation cohort and 780 in the postimplementation cohort; 202 patients had methadone restarts before implementation (464 restart episodes; 124 male [61.4%]; 148 ages 25 to 44 years [73.3%]; 10 Black [5.0%], 72 Hispanic [35.6%], 112 White [55.4%]) and 195 patients after implementation (489 restart episodes; 116 male [59.5%]; 123 ages 25 to 44 years [63.1%]; 12 Black [6.2%], 60 Hispanic [30.8%], 114 White [58.5%]). Most patients were insured by Medicaid (preimplementation, 164 of 202 patients [81.2%]; postimplementation, 154 of 195 patients [79.0%]). In the preimplementation period, restart doses were 32.8% lower (95% CI, 30.8%-34.7%) than the last dose prior to restart. After protocol implementation, restart doses were 3.4% lower (95% CI, 1.5%-5.3%) than the last received dose. In the preimplementation period, 44 of 464 restarts (9.5%) were followed by ED visits within 7 days, compared with 30 of 489 restarts (6.1%) in the postimplementation period (aRR, 0.61; 95% CI, 0.37-0.98). There were 4 restarts followed by death within 90 days in both the preimplementation and postimplementation periods. Treatment retention was similar in preimplementation and postimplementation periods (aRR, 0.88; 95% CI, 0.73-1.05). Survey responses indicated high acceptance and satisfaction with the protocol.

**Conclusions and Relevance:**

In this cohort study of individuals with opioid use disorder restarting methadone after a gap in treatment, an individualized methadone restart protocol that considered interim opioid tolerance was associated with higher restart doses without compromising safety or treatment retention. Further research is needed to evaluate if this approach improves treatment outcomes.

## Key Points

**Question:**

Are individualized higher methadone restart doses after a gap in daily dosing associated with safety and effectiveness?

**Findings:**

In this cohort study, a new clinical protocol that individualized methadone restart doses by accounting for maintenance of opioid tolerance after a gap in methadone treatment was associated with significantly higher restart doses without any changes in opioid-related harms or treatment retention.

**Meaning:**

These results suggest that more individualized methadone restart dosing that accounts for maintenance of opioid tolerance may promote higher restart doses that are closer to therapeutic levels without increased patient harm.

## Teaser

This cohort study of US adults receiving methadone to treat opioid use disorders examines changes in restart doses and health outcomes following the adoption of an individualized methadone restart protocol.

## Introduction

Methadone is a highly effective medication for opioid use disorder that is associated with a 50% reduction in mortality.^1,2^ Due to federal regulations, methadone may only be administered in licensed opioid treatment programs (OTP), where patients are frequently required to present for daily in-person dosing.^3^ In OTPs, methadone restarts, where individuals miss multiple days of dosing and then return to care, are common and typically result in methadone dose reductions due to presumed loss of opioid tolerance. Lower methadone doses may be associated with clinical instability and risk of dropout from care.^4^ In determining appropriate restart doses, OTP clinicians must weigh the benefits of maintaining a more therapeutic methadone dose against the risks of overmedication or overdose from interval loss of opioid tolerance. The Substance Abuse and Mental Health Services Administration (SAMHSA) consensus documents do not explicitly dictate the dose after missed days.^5,6^

In the absence of evidence-based guidelines on methadone restart dosing, clinicians rely on consensus best practice documents, which generally recommend substantial dose decreases after as few as 3 missed days of dosing.^5,7,8,9^ For example, SAMHSA’s Treatment Improvement Protocol 63 states, “Patients who miss more than 4 doses must be reassessed. Their next methadone dose should be decreased substantially and built back up gradually. It may be necessary to restart the dose induction process from Day 1.”^5^ These recommendations are based on pharmacologic concepts of tolerance, yet most do not distinguish between individuals with loss of tolerance from opioid abstinence vs maintenance of tolerance from interim use of other prescribed or non-prescribed opioids. This approach can lead to substantial underdosing of opioid tolerant individuals, resulting in ongoing illicit opioid use to alleviate withdrawal symptoms and further clinical instability. Furthermore, this uniform approach ignores patient preference, clinician discretion, and shared decision-making based on informed risk-benefit discussions, the importance of which is highlighted in SAMHSA’s February 2024 update to 42 CFR Part 8 regulations.^3^ The arrival of illicitly manufactured fentanyl in the opioid supply has spurred a re-examination of methadone dose initiation and titration.^10,11,12,13^ However, only recently has restart dosing garnered attention,^3^ and limited data exist to guide modifications to restart dosing practices.

In 2022, our safety-net hospital-based OTP implemented a novel methadone restart protocol. The new protocol utilized a clinical, rather than rule-based, model as the basis for restart dosing and included a risk-benefit discussion and an individualized assessment that accounted for interval opioid use. In this study, we present findings from a retrospective evaluation of this novel protocol focusing on patient safety, acceptability, and retention in treatment.

## Methods

Denver Health and Hospital Authority (DH) is a comprehensive, integrated safety-net health system located in Denver, Colorado, that includes an OTP with a census of approximately 750 patients. This project was reviewed by the Denver Health Quality Improvement Review Committee, authorized by the Colorado Multiple Institutional Review Board at the University of Colorado Denver, and was determined to be exempt from informed consent requirements because it was not human participants research. We followed the Strengthening the Reporting of Observational Studies in Epidemiology (STROBE) reporting guideline for cohort studies.

### Intervention

The DH OTP restart process requires the OTP clinician to enter a new medication order after 4 or more days of missed doses. Prior to 2022, protocol at the OTP was to decrease a patient’s methadone dose by 50% after missing 4 to 6 days, and to restart dosing at 30 mg or less after missing 7 or more days. Between February and November 2022, following trials with individual patients, the OTP implemented and refined a new restart protocol that evaluated patients’ interim opioid use through direct patient report, urine toxicology, and/or signs and symptoms of intoxication or withdrawal (Figure). Grounded in the principles of opioid cross-tolerance^14,15,16^ and prior research indicating patients often underreport opioid use,^17,18,19,20^ the new restart protocol recommended substantially smaller dose reductions for patients reporting interval nonprescribed opioid use sufficient to prevent or alleviate withdrawal symptoms.

**Figure.**
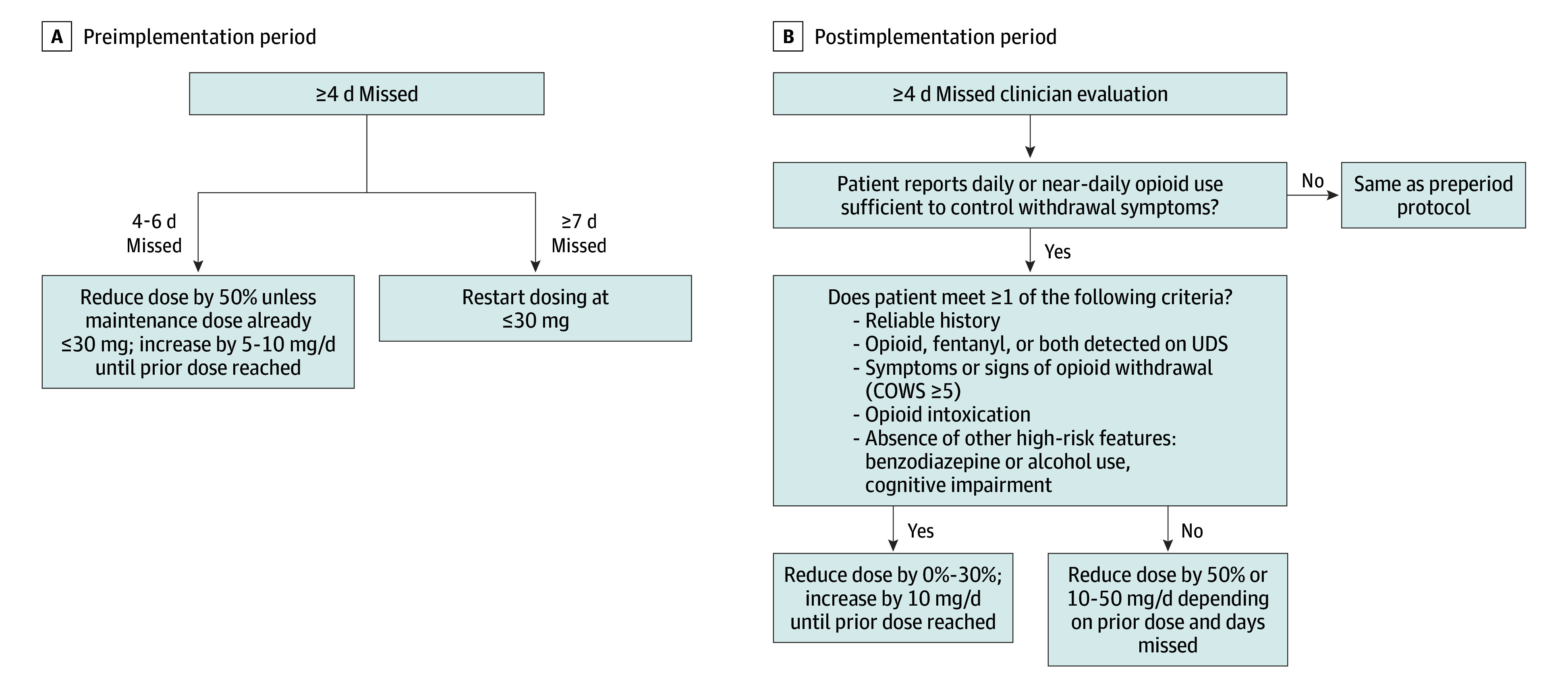
Methadone Restart Protocol at the Denver Health Opioid Treatment Program Before and After 2022 Changes COWS indicates Clinical Opiate Withdrawal Scale; UDS, urine drug stream.

### Study Sample and Data Sources

Following full implementation of the new protocol in November 2022, we performed a retrospective preimplementation and postimplementation study to estimate the association of the new restart protocol with patient safety and treatment retention. The preimplementation period included all restart episodes from January 1, 2021, to December 31, 2021, while the postimplementation period spanned January 1, 2023, to December 31, 2023. From January through March 2024, we also gathered a convenience sample of patient surveys to assess patient perspectives on restart tolerability and satisfaction, and surveyed clinicians on perceptions of safety and acceptability.

Data on restart methadone doses and number of missed days were extracted from the Substance Abuse and Medication Management System (SAMMS). Patients in SAMMS were linked to DH’s systemwide electronic health record (EHR) (Epic) to enable collection of covariate and outcome data. Methadone restarts were identified as the first dose after 4 or more days of missed doses (up to 30 days, as more than 30 days of missed dosing results in an automatic discharge from the OTP). Patients could be included in both preimplementation and postimplementation periods and could have multiple restarts in the same period.

### Outcomes

The primary outcome was patient safety, defined as DH emergency department (ED) visits within 7 days after a restart and all-cause mortality within 7 and 90 days after a restart. We also evaluated ED visits for opioid withdrawal or drug overdose within 7 days after a restart. We chose both 7- and 90-day all-cause mortality to balance the need for statistical power while still assessing a clinically relevant treatment period during which the risk of death is higher.^21,22^ The secondary outcome was 90-day treatment retention, defined as no continuous gap in dosing greater than 30 days. We chose a 30-day gap because our OTP keeps patients enrolled for up to 30 days during which time outreach efforts are made to try to re-engage the patient in treatment. Our process outcome was percentage change from the last dose before a patient missed doses to the dose at restart.

For ED visits, we evaluated all-cause visits as well as visits related to opioid withdrawal (*International Statistical Classification of Diseases, Tenth Revision, Clinical Modification [ICD-10-CM]* code starting with “F11” and EHR diagnosis name containing “withdrawal”) or drug overdose (*ICD-10-CM* codes starting with “T40” and EHR diagnosis name containing “overdose”). For all-cause mortality, state death certificates are regularly matched to the DH EHR as described previously.^23^ Patients with a date of death within 7 or 90 days after a restart were captured and the underlying cause of death was noted. For 90-day retention and restart doses, we used SAMMS data to calculate the number of missed dosing days and dose strength (in mg) immediately preceding and following a gap in treatment of more than 4 days and less than 30 days.

### Covariates

Demographic variables included self-reported race, ethnicity, and legal sex. Race and ethnicity were included as markers of social discrimination and because they have been associated with methadone initiation and retention. Age at first restart in the period and insurance type within 14 days of the patient’s first restart in the period were extracted from the EHR. A patient was considered to have experienced homelessness if they were included in DH’s EHR homelessness registry during the preimplementation or postimplementation period. As described previously, this registry uses multiple markers within the EHR, beyond address and registration data, to identify homelessness.^24^ Comorbid stimulant use disorder and alcohol use disorder (AUD) were based on diagnosis codes, patient-reported screening tools, laboratory tests, and other markers of use disorders in the EHR in the period of interest (eText in Supplement 1). Other co-occurring use disorders were identified by encounter or problem list *ICD-10-CM* codes starting with F13 (sedatives), F14 (cocaine), F16 (hallucinogens), F18 (inhalants), and F19 (other) (eTable 1 in Supplement 1).

### Statistical Analysis

We calculated frequencies and proportions of patient demographics and methadone restart characteristics, stratified by preimplementation and postimplementation periods. Covariate balance between periods was assessed using standardized mean differences (SMDs) with a pooled standard deviation. Means and standard deviations of doses before and at restart were computed. We used a linear mixed-effects model to assess average individual-level percentage changes in methadone dose before and at restart comparing preimplementation and postimplementation periods and accounting for clustering within individuals with multiple restarts. We performed additional analyses stratified by missing less than or greater than 7 days of dosing. Statistically significant differences in doses were determined by 95% CIs and 2-sided *P* < .05.

To estimate the association of the new restart policy with changes in outcomes, we used generalized estimating equations (GEE). The GEE approach was chosen to account for repeated observations within individuals, as all restart episodes, including overlapping episodes within individuals, were included in analyses. A log-binomial model was selected to estimate risk ratios (RRs) and 95% confidence intervals, and we specified an exchangeable correlation structure. All models were adjusted for sex; race and ethnicity (Black or African American; Hispanic, Latino, or Spanish origin; White; and other); age category; insurance type; presence of stimulant use disorder, AUD, or other use disorders; and housing status. All analyses were performed in SAS Enterprise Guide version 8.3 (SAS Institute Inc) and R version 4.1.3 (R Foundation for Statistical Computing) and were completed August 2024 through June 2025.

### Patient and Clinician Experience Survey

Between January and March 2024, a convenience sample of patients completed a 6-question paper survey about the new methadone restart protocol. Surveys were anonymous, advertised by a flyer in the OTP, and collected in a locked box. Surveys asked about withdrawal and sedation following restart and patient satisfaction with the restart dose. During this same time, a 9-question electronic survey was administered via REDCap^25^ to prescribing OTP clinicians assessing trust in patient reports, reliance on objective data to determine opioid tolerance, perceived safety, and acceptability of higher restart doses. No compensation was provided to patients or clinicians for survey participation.

## Results

A total of 202 of 786 total OTP patients (25.7%) had restarts in the preimplementation period (464 restart episodes; 124 male [61.4%]; 148 ages 25 to 44 years [73.3%]; 10 Black [5.0%], 72 Hispanic [35.6%], 112 White [55.4%]), and 195 of 780 total OTP patients (25.0%) had restarts in the postimplementation period (489 restart episodes; 116 male [59.5%]; 123 ages 25 to 44 years [63.1%]; 12 Black [6.2%], 60 Hispanic [30.8%], 114 White [58.5%]) (Table 1). Most patients in both cohorts were insured by Medicaid (preimplementation, 164 of 202 patients [81.2%]; postimplementation, 154 of 195 patients [79.0%]). Over half of patients (99 of 195 [50.8%]) were unhoused in the postimplementation period compared with 82 of 202 patients (40.6%) in the preimplementation period, and the majority in both cohorts had a co-occurring stimulant use disorder (preimplementation, 158 of 202 [78.2%]; postimplementation, 164 of 195 patients [84.1%]). The distribution of the number of methadone restarts per individual in each period was similar.

**Table 1.** Descriptive Characteristics of Patients With Methadone Restarts Before and After New Restart Protocol

Characteristics	Patients, No. (%)		Standardized mean difference (SMD)^a^
	Preimplementation cohort (2021) (n = 786)	Postimplementation cohort (2023) (n = 780)	
Patients with restarts	202 (25.7)	195 (25.0)	
Sex			
Male	124 (61.4)	116 (59.5)	0.04
Female	78 (38.6)	79 (40.5)	
Age at first restart, y			
18-24	9 (4.5)	12 (6.2)	0.36
25-34	81 (40.1)	50 (25.6)	
35-44	67 (33.2)	73 (37.4)	
45-54	21 (10.4)	32 (16.4)	
55-64	19 (9.4)	17 (8.7)	
≥65	5 (2.5)	11 (5.6)	
Race and ethnicity			
Black or African American	10 (5.0)	12 (6.2)	0.11
Hispanic, Latino, or Spanish origin	72 (35.6)	60 (30.8)	
White	112 (55.4)	114 (58.5)	
Other or unknown race^b^	8 (4.0)	9 (4.6)	
Insurance within 14 d of first restart			
Medicaid	164 (81.2)	154 (79.0)	0.16
Medicare	16 (7.9)	24 (12.3)	
Commercial	10 (5.0)	7 (3.6)	
Other	12 (5.9)	10 (5.1)	
Housing status			
Housed for entire period	120 (59.4)	96 (49.2)	0.21
Unhoused at any point in the period	82 (40.6)	99 (50.8)	
Co-occurring SUD			
Stimulant use disorder	158 (78.2)	164 (84.1)	0.15
Alcohol use disorder	47 (23.3)	38 (19.5)	0.09
Other substance use disorder	101 (50.0)	91 (46.7)	0.07
No. of restarts			
1	101 (50.0)	97 (49.7)	0.12
2	48 (23.8)	40 (20.5)	
3-4	31 (15.3)	30 (15.4)	
≥5	22 (10.9)	28 (14.4)	

Abbreviation: SUD, substance use disorder.

^a^ Standardized mean difference calculated using pooled standard deviations across groups.

^b^ Other included American Indian and Alaska Native, Asian, Native Hawaiian and other Pacific Islander, “other race” selected by the patient, and multiracial.

### Restart Dosing

During the preimplementation period, the mean (SD) methadone dose immediately prior to restart was 74 (45) mg among 464 restarts (Table 2). The mean (SD) dose at restart was 47 (34) mg, representing a 32.8% reduction (95% CI, 30.8% to 34.7%) as estimated from the linear mixed model. Those missing fewer than 7 days had a 27.5% reduction (95% CI, 25.2% to 29.8%) in their dose, and those missing 7 or more days had a 38.2% reduction (95% CI, 35.0% to 41.5%) in their dose (eTable 2 in Supplement 1). During the postimplementation period, the mean (SD) methadone dose immediately prior to restart was 100 (42) mg among 489 restarts (Table 2). The mean (SD) dose at restart was 95 (42) mg, representing a 3.4% reduction (95% CI, 1.5% to 5.3%) as estimated from the linear mixed model. Those missing fewer than 7 days had a 0.9% increase (95% CI, −1.4% to 3.3%) in dose, and those missing 7 or more days had a 6.6% reduction (95% CI, −9.7% to 3.5%) in their dose (eTable 2 in Supplement 1). The reduction in restart dose was significantly less after implementation of the new restart protocol (*P* < .001 in the linear mixed model).

**Table 2.** Methadone Restart Episode Characteristics and Changes in Dosing During the Preimplementation and Postimplementation Periods for the New Restart Protocol

Characteristics	Preimplementation period (2021)	Postimplementation period (2023)
No. of restart episodes	464	489
No. of missed dosing days, median (IQR)	7 (5 to 12)	7 (5 to 11)
Methadone dose prior to restart, mean (SD), mg	74 (45)	100 (42)
Methadone dose at restart, mean (SD), mg	47 (34)	95 (42)
Modeled change in methadone dose at restart, % (95% CI)^a^	−32.8 (−34.7 to −30.8)	−3.4 (−5.3 to −1.5)

^a^ Statistical test for differences in average individual-level percentage change comparing preimplementation and postimplementation periods comes from a linear mixed model controlling for clustering at the patient level to account for individuals with multiple restarts.

### Patient Safety

In the 7 days after a methadone restart, there were 44 all-cause ED visits in the preimplementation period (9.5% of all restarts) and 30 all-cause ED visits in the postimplementation period (6.1% of all restarts) (Table 3). In fully adjusted GEE models, the risk of an ED visit in the 7 days after a methadone restart was 39% lower in the postimplementation period (aRR, 0.61; 95% CI, 0.37-0.98). ED visits specifically for opioid withdrawal and/or drug overdose within 7 days of a restart were rare in both the preimplementation (3 cases) and postimplementation periods (no cases). For all-cause mortality, there were 2 deaths (1 from overdose) in the preimplementation period and 1 death (not from overdose) in the postimplementation period within 7 days of methadone restart, and 4 deaths (2 from overdose) in the preimplementation period and 4 deaths (zero from overdose) in the postimplementation period within 90 days of methadone restart.

**Table 3.** Association of New Methadone Restart Protocol With ED Visits Within 7 Days After Restart, All-Cause Mortality, and 90-Day Treatment Retention

Outcomes	Patients, No. (%)		Risk ratio (95% CI)	
	Preimplementation (2021) (n = 464)	Postimplementation (2023) (n = 489)	Unadjusted	Adjusted^a^
ED visit for any cause within 7 d after restart	44 (9.5)	30 (6.1)	0.65 (0.39-1.09)	0.61 (0.37-0.98)
ED visit for opioid withdrawal or drug overdose within 7 d after restart^b^	3 (0.6)	0	NA	NA
All-cause mortality within 7 d after restart^b^	2 (0.4)	1 (0.2)	NA	NA
All-cause mortality within 90 d after restart^b^	4 (0.9)	4 (0.8)	NA	NA
Treatment retention at 90 d after restart	242 (52.2)	253 (51.7)	0.88 (0.73-1.07)	0.88 (0.73-1.05)

Abbreviations: ED, emergency department; NA, not applicable.

^a^ Fully adjusted model controls for age, sex, race and ethnicity, insurance status, housing status, and co-occurring substance use disorders. Adjusted risk ratios are from generalized estimating equations using log-binomial distribution and exchangeable correlation matrix.

^b^ For the outcomes of ED visits for opioid withdrawal or drug overdose within 7 days after restart and all-cause mortality within 7 and 90 days after restart, adjusted models were not attempted due to the low frequency of outcomes.

### Treatment Retention

Retention in methadone treatment at 90-day after restart was approximately half in both the preimplementation (242 of 464 [52.2%]) and postimplementation cohorts (253 of 489 [51.7%]) (Table 3). In fully adjusted GEE models, there was no association between the new restart protocol and treatment retention (aRR, 0.88; 95% CI, 0.73-1.05). Overall, 90-day retention at the OTP for all patients (including those with and without restarts) was 68% in the preimplementation period and 64% in the postimplementation period.

### Patient and Clinician Experiences

Twenty-one patients completed the anonymous survey. Of the 11 patients who reported no dose change after treatment interruption, 10 (91%) reported satisfaction with their restart dose, citing reasons of autonomy and stability. Four of 9 (44%) patients who reported a lower restart dose reported satisfaction with their restart dose (full results in eTable 3 in Supplement 1). Of the 6 clinicians who completed the survey, all 6 (100%) strongly agreed that starting an opioid-tolerant patient back on a methadone dose at or near the prior dose is safe, satisfactory to staff and patients, and encourages ongoing retention (eTable 4 in Supplement 1).

## Discussion

In this analysis of a novel methadone restart protocol that accounts for interim opioid tolerance during missed dosing days, we found that this patient-centered protocol was associated with significantly higher restart doses that were closer to patients’ last received dose, without associated harms to patient safety. We also found no association between the new restart protocol and 90-day treatment retention. The acceptance of more risk from faster titration in exchange for more rapid stabilization, while also considering the risk of underdosing, reflects a changing paradigm that we applied to our restart process.

The new methadone restart protocol was associated with considerable changes in dosing practices at the OTP. While methadone doses were generally higher in the postimplementation period relative to the preimplementation period, likely due to further fentanyl saturation in the local illicit opioid market,^26^ it is noteworthy that in the postimplementation period, methadone restart doses were much closer to doses prior to restart, decreasing by only 3.4% compared with 32.8% in the preimplementation period. Changes in restart doses for patients missing 7 or more days were the most stark, with restart doses being an average of 6.6% lower in the postimplementation period compared with 38.2% lower in the preimplementation period. Despite the higher dosing strategy, implementing the new protocol was not associated with worse patient safety outcomes, although overdose outcomes were rare in both periods. ED visits within 7 days of restart were lower after the new protocol’s implementation, and there were zero overdose deaths within 7 or 90 days of a restart. Higher restart doses were not associated with a change in 90-day treatment retention, suggesting that a more therapeutic restart dose is only 1 component of successfully retaining patients in care. However, methadone treatment retention in Colorado was declining during the study period,^27^ and the stable retention in our study may point toward a potential retention benefit that should be evaluated in future studies. Taken together, these results suggest that more patient-centered methadone restart dosing that considers individual patient tolerance and preferences is likely to be safe.

Current consensus best practice documents, implemented by most OTPs, recommend substantial methadone dose reductions to avoid harm.^5^ However, when restarting at half or less of the previous methadone dose, some patients may lose hope of regaining access to previously therapeutic doses, leading them to disengage in care and initiating a downward spiral. The efforts required to obtain illicit opioids to alleviate severe withdrawal symptoms may result in additional missed dosing days, further dose reductions, and treatment discontinuation. Retention in treatment is life-preserving,^28^ so this potential iatrogenic destabilization should be avoided when possible. OTP clinicians and directors can weigh methadone safety against the risks of underdosing, patient experience, and return to illicit opioid use. While our novel restart protocol was not associated with clear harms or improvements in retention, additional multisite studies that are powered to evaluate rare events are needed to help OTPs weigh the risks and benefits of implementing higher restart doses.

### Limitations

There are several important limitations to this study’s findings. First, this is an observational, pre-post study that lacks an external control group. As such, differences in our outcomes may not be solely caused by the new restart protocol. Second, while our restart protocol prior to the 2022 change indicated clinicians should reduce methadone doses by at least 50% after 4 or more missed dosing days, observed decreases (33%) were smaller. As such, many clinicians were already using higher methadone restart doses in the period prior to implementation than are often recommended in consensus statements, and thus the effect of our new protocol on treatment outcomes may be more muted. Third, given that ED visits for nonfatal overdose and all-cause mortality are rare events, this study is likely underpowered to detect small yet clinically meaningful safety outcomes.^29^ A significant proportion of nonfatal overdoses are managed in the community by bystanders and emergency medical services (EMS) without transport to the ED, which may have led to underascertainment of this outcome.^30,31^ Furthermore, we only had ED data from a single health system and would have missed ED visits at other health systems. Future studies using larger sample sizes with data linked across health systems and EMS encounters can further help clarify the safety of higher methadone restart doses. Fourth, we were unable to systematically assess ongoing illicit substance use after methadone restart, which may be of interest in future studies. Fifth, our patient survey was taken from a convenience sample, and patients with more extreme opinions of higher methadone restarts may have been more likely to respond, making their responses less representative.

## Conclusions

In this cohort study of individuals with opioid use disorder restarting methadone after a gap in treatment, an individualized methadone restart protocol that relied on patient self-report and clinician assessments of interval opioid use and maintenance of tolerance was associated with higher restart doses without compromising safety or treatment retention. Our findings suggest that traditional methadone restart protocols that recommend substantial dose reductions may fail to address important individual heterogeneity in restart dosing needs, although additional studies powered to detect small changes in safety outcomes are needed.

## Notes

Supplement 1. **eAppendix.** Case Definition for Comorbid Stimulant Use Disorder and Alcohol Use Disorder **eTable 1.** *ICD-10-CM* Codes Used for Identifying Comorbid Substance Use Disorders **eTable 2.** Changes in Methadone Doses From Before to at Restart by Pre- and Postperiod for the New Restart Protocol, Overall and Stratified by Duration of Gap in Methadone Dosing **eTable 3.** Patient Satisfaction Survey Responses **eTable 4.** Clinician Survey Responses Regarding Perceived Acceptability and Safety of Restart Protocol

Supplement 2.
Data Sharing Statement
