# Supplementary material for: Evaluation of a Novel Patient-Centered Methadone Restart Protocol
Source: JAMA Netw Open. 2025 Aug 28;8(8):e2529393. doi: 10.1001/jamanetworkopen.2025.29393 (PMC12395315; doi:10.1001/jamanetworkopen.2025.29393)
Supplement: Supplement 1. — eAppendix. Case Definition for Comorbid Stimulant Use Disorder and Alcohol Use Disorder eTable 1. ICD-10-CM Codes Used for Identifying Comorbid Substance Use Disorders eTable 2. Changes in Methadone Doses From Before to at Restart by Pre- and Postperiod for the New Restart Protocol, Overall and Stratified by Duration of Gap in Methadone Dosing eTable 3. Patient Satisfaction Survey Responses eTable 4. Clinician Survey Responses Regarding Perceived Acceptability and Safety of Restart Protocol [file jamanetwopen-e2529393-s001.pdf]

Supplemental Online Content

Christine PJ, Blum J, Tillman AR, et al. Evaluation of a novel patient-centered methadone restart protocol. *JAMA Netw Open.* 2025;8(8):e2529393. doi:10.1001/jamanetworkopen.2025.29393

eAppendix. Case Definition for Comorbid Stimulant Use Disorder and Alcohol Use Disorder

eTable 1. *ICD-10-CM* Codes Used for Identifying Comorbid Substance Use Disorders

eTable 2. Changes in Methadone Doses From Before to at Restart by Pre- and Postperiod for the New Restart Protocol, Overall and Stratified by Duration of Gap in Methadone Dosing

eTable 3. Patient Satisfaction Survey Responses

eTable 4. Clinician Survey Responses Regarding Perceived Acceptability and Safety of Restart Protocol

This supplemental material has been provided by the authors to give readers additional information about their work.

© 2025 Christine PJ et al. *JAMA Network Open*.

eText 1: Case definition for comorbid stimulant use disorder and alcohol use disorder

Comorbid stimulant use disorder was identified if an individual met one or more of the following criteria:

1) an ICD-10-CM code on an encounter or active in the problem list starting with F15 or otherwise known to involve amphetamines;
2) a urine drug screen positive for a methamphetamine analyte on confirmatory testing;
3) a positive screen for amphetamine use (i.e. an Alcohol, Smoking and Substance Involvement Screening Test (ASSIST) amphetamine score greater than zero);
4) a referral, intake, or active episode of substance use disorder (SUD) treatment concerning stimulant use;
5) disclosed methamphetamine use on a SUD history screen

Similarly, co-occurring alcohol use disorder (AUD) was identified if an individual met one or more of the following criteria:

1) an ICD-10-CM code on an encounter or active in the problem list starting with F10 (excluding remission codes);
2) a positive screen for AUD (i.e. an Alcohol Use Disorders Identification Test-Concise (AUDIT-C) score of 4 or more for men or 3 or more for women or an ASSIST alcohol score over 11 ;
3) a non-zero alcohol withdrawal scale (e.g. Severity of Ethanol Withdrawal Scale (SEWS) score;
4) a medication order, fill, or administration of medication for AUD or a medication for alcohol withdrawal including: naltrexone, disulfiram, or acamprosate order with ICD-10-CM starting with F10 on the order or on associated encounter, OR topiramate, gabapentin, phenobarbital, clorazepate, diazepam, carbamazepine, or chlordiazepoxide with an ICD-10-CM code starting with F10 on the order or the only code on the associated encounter

© 2025 Christine PJ et al. *JAMA Network Open*.

eTable 1. ICD-10-CM codes used for identifying comorbid substance use disorders and ED visits for opioid withdrawal and overdose

Alcohol use disorder codes	F10.10, F10.120, F10.121, F10.129, F10.14, F10.150, F10.151, F10.159, F10.180, F10.182, F10.188, F10.19, F10.20, F10.220, F10.221, F10.229, F10.24, F10.250, F10.251, F10.259, F10.26, F10.27, F10.280, F10.281, F10.282, F10.288, F10.29, F10.90, F10.920, F10.921, F10.929, F10.94, F10.950, F10.951, F10.959, F10.96, F10.97, F10.980, F10.982, F10.988, F10.99
Stimulant use disorder codes	F15.10, F15.11, F15.120, F15.121, F15.122, F15.129, F15.13, F15.14, F15.150, F15.151, F15.159, F15.180, F15.182, F15.188, F15.19, F15.20, F15.21, F15.220, F15.221, F15.222, F15.229, F15.23, F15.24, F15.250, F15.251, F15.259, F15.280, F15.282, F15.288, F15.29, F15.90, F15.91, F15.920, F15.921, F15.922, F15.929, F15.93, F15.94, F15.950, F15.951, F15.959, F15.980, F15.981, F15.982, F15.988, F15.99, T43.621A, T43.621D, T43.621S, T43.622A, T43.622D, T43.622S, T43.623A, T43.623D, T43.624A, T43.624D, T43.624S, T43.625A, T43.625D, T43.625S, T43.651A, T43.654D, T43.654S
Other use disorder codes: Sedatives	F13.10, F13.120, F13.121, F13.129, F13.130, F13.131, F13.132, F13.139, F13.14, F13.150, F13.159, F13.180, F13.188, F13.19, F13.20, F13.220, F13.221, F13.229, F13.230, F13.231, F13.232, F13.239, F13.24, F13.250, F13.251, F13.259, F13.280, F13.282, F13.288, F13.29, F13.90, F13.920, F13.921, F13.929, F13.930, F13.931, F13.932, F13.939, F13.94, F13.951, F13.959, F13.980, F13.982, F13.988, F13.99
Other use disorder codes: Cocaine	F14.10, F14.120, F14.121, F14.122, F14.129, F14.13, F14.14, F14.150, F14.151, F14.159, F14.180, F14.182, F14.188, F14.19, F14.20, F14.220, F14.221, F14.222, F14.229, F14.23, F14.24, F14.250, F14.251, F14.259, F14.280, F14.282, F14.288, F14.29, F14.90, F14.920, F14.921, F14.922, F14.929, F14.93, F14.94, F14.950, F14.951, F14.959, F14.980, F14.982, F14.988, F14.99
Other use disorder codes: Hallucinogens	F16.10, F16.120, F16.121, F16.122, F16.129, F16.14, F16.150, F16.151, F16.159, F16.180, F16.183, F16.188, F16.19, F16.20, F16.220, F16.221, F16.229, F16.24, F16.250, F16.251, F16.259, F16.280, F16.283, F16.288, F16.29, F16.90, F16.920, F16.921, F16.929, F16.94, F16.950, F16.951, F16.959, F16.980, F16.983, F16.988, F16.99
Other use disorder codes: Inhalants	F18.10, F18.120, F18.121, F18.129, F18.14, F18.150, F18.151, F18.159, F18.180, F18.19, F18.20, F18.229, F18.24, F18.251, F18.259, F18.288, F18.29, F18.90, F18.929, F18.94, F18.959, F18.980, F18.988, F18.99
Other use disorder codes: Other	F19.10, F19.120, F19.121, F19.122, F19.129, F19.130, F19.131, F19.132, F19.139, F19.14, F19.150, F19.151, F19.159, F19.180, F19.182, F19.188, F19.19, F19.20, F19.220, F19.221, F19.222, F19.229, F19.230, F19.231, F19.232, F19.239, F19.24, F19.250, F19.251, F19.259, F19.27, F19.280, F19.282, F19.288, F19.29, F19.90, F19.920, F19.921, F19.922, F19.929, F19.930, F19.931, F19.932, F19.939, F19.94, F19.950, F19.951, F19.959, F19.96, F19.97, F19.980, F19.981, F19.982, F19.988, F19.99

© 2025 Christine PJ et al. *JAMA Network Open*.

Opioid withdrawal (code + 'WITHDRAWAL' in EHR diagnosis name)	F11.10, F11.11, F11.120, F11.121, F11.122, F11.129, F11.13, F11.14, F11.151, F11.159, F11.182, F11.188, F11.19, F11.20, F11.21, F11.220, F11.221, F11.222, F11.229, F11.23, F11.24, F11.250, F11.251, F11.259, F11.281, F11.282, F11.288, F11.29, F11.90, F11.91, F11.920, F11.921, F11.922, F11.929, F11.93, F11.94, F11.950, F11.951, F11.959, F11.981, F11.982, F11.988, F11.99
Opioid overdose (code + 'OVERDOSE' in EHR diagnosis name)	T40.0X1A, T40.0X2A, T40.0X4A, T40.0X4D, T40.0X5A, T40.0X5D, T40.1X1A, T40.1X1D, T40.1X1S, T40.1X2A, T40.1X2D, T40.1X2S, T40.1X3D, T40.1X4A, T40.1X4D, T40.1X4S, T40.2X1A, T40.2X1D, T40.2X1S, T40.2X2A, T40.2X2D, T40.2X2S, T40.2X3A, T40.2X4A, T40.2X4D, T40.2X4S, T40.2X5A, T40.2X5D, T40.2X5S, T40.2X6A, T40.3X1A, T40.3X1D, T40.3X1S, T40.3X2A, T40.3X2D, T40.3X4A, T40.3X4D, T40.3X5A, T40.3X5D, T40.3X5S, T40.3X6A, T40.411A, T40.411D, T40.411S, T40.412A, T40.412D, T40.413A, T40.414A, T40.414D, T40.415A, T40.415D, T40.416A, T40.421A, T40.422A, T40.422D, T40.422S, T40.425A, T40.425D, T40.425S, T40.491A, T40.492A, T40.492D, T40.494A, T40.495A, T40.495S, T40.496A, T40.4X1A, T40.4X2A, T40.4X2D, T40.4X4A, T40.4X5A, T40.4X5D, T40.4X6A, T40.5X1A, T40.5X1D, T40.5X1S, T40.5X2A, T40.5X2D, T40.5X3A, T40.5X4A, T40.5X4D, T40.5X4S, T40.5X5A, T40.5X5D, T40.5X5S, T40.601A, T40.601D, T40.601S, T40.602A, T40.602D, T40.602S, T40.603A, T40.604A, T40.604D, T40.604S, T40.605A, T40.605D, T40.691A, T40.691D, T40.692A, T40.694A, T40.695A, T40.711A, T40.711D, T40.711S, T40.712A, T40.712D, T40.714A, T40.714S, T40.715A, T40.715S, T40.721A, T40.722A, T40.725A, T40.7X1A, T40.7X1D, T40.7X1S, T40.7X2A, T40.7X2D, T40.7X4A, T40.7X5A, T40.7X5S, T40.8X1A, T40.8X1D, T40.8X1S, T40.8X2A, T40.8X3A, T40.8X4A, T40.8X4S, T40.901A, T40.902A, T40.904A, T40.905A, T40.991A, T40.991D, T40.992A, T40.994A, T40.995A

© 2025 Christine PJ et al. *JAMA Network Open*.

eTable 2: Changes in methadone doses from before to at restart by pre- and post-period for the new restart protocol, overall and stratified by duration of gap in methadone dosing

Dosing gap	Pre-period			Post-period			
	Dose prior to restart, mean mg	Dose at restart, mean mg	Modeled percent change in dose at restart (95% CI)[a]	Dose prior to restart, mean mg	Dose at restart, mean mg	Modeled percent change in dose at restart (95% CI)[a]	Difference in percent change in dose at restart comparing pre- and post-period (95% CI)[b]
**Overall**	74	47	-32.8 (-34.7, -30.8)	100	95	-3.4 (-5.3, -1.5)	29.4 (26.6, 32.1)[c]
**< 7 Days**	78	56	-27.5 (-29.8, -25.2)	102	101	0.9 (-1.4, 3.3)	28.4 (25.2, 31.7)[c]
**≥ 7 Days**	70	39	-38.2 (-41.5, -35.0)	98	90	-6.6 (-9.7, -3.5)	31.6 (27.2, 36.1)[c]

[a] Model estimates represent average individual-level percent change comparing doses prior to restart and at restart. Estimates are from a linear mixed model controlling for clustering at the patient-level to account for individuals with multiple restarts.
[b] Statistical test for differences in percent change comparing pre- and post-periods come from linear mixed model controlling for clustering at the patient-level to account for individuals with multiple restarts.
[c] $p<0.0001$

© 2025 Christine PJ et al. *JAMA Network Open*.

eTable 3. Patient satisfaction survey responses[a]

Question	Responses (n = 21), No. (%)	
How did your restart dose compare to your previous dose?	Lower	9 (42.8)
	The same	11 (52.3)
	Higher	1 (4.8)
The dose given to me at restart was	Too high	0 (0)
	About right	9 (42.8)
	Too low	9 (42.8)
	Unsure	3 (14.3)
Overall, how satisfied were you with the methadone dose given at restart?	Very unsatisfied	3 (14.3)[b]
	Slightly unsatisfied	3 (14.3)[b]
	Slightly satisfied	6 (28.5)
	Very satisfied	9 (42.8)

Patient comments regarding restarts

1. "I would not want my dose to change just because I did a restart."
2. "I really appreciated not having to start at 50 mg or having to go through the whole process every time."
3. "You all were amazing getting me back into the clinic quickly and respectfully. Coming back was hard, but ALL of the staff were fantastic. Smooth reentry."
4. "I highly appreciate not having to start at a low dose because that means supplementing w/ street drugs"
5. "Amazing staff, quickly restarted with no judgement, lots of compassion, understanding and care. Thanks."

[a] Survey responses come from a convenience sample of patients who elected to fill out the survey at the OTP.
[b] Of the 6 patients who reported being slightly or very unsatisfied with the restart dose, 5 were restarted on a dose lower than their previous dose.

© 2025 Christine PJ et al. *JAMA Network Open*.

eTable 4: Clinician survey responses regarding perceived acceptability and safety of restart protocol

Question	Moderately or strongly agree, No. (%)	Moderately or strongly disagree, No. (%)
I feel comfortable starting a patient who has maintained opioid tolerance on a methadone dose at or close to their prior methadone dose. (n = 6)	6 (100)	0 (0)
Starting a tolerant client back on a methadone dose at or near the prior methadone dose is safe. (n = 6)	6 (100)	0 (0)
Starting a tolerant client back on a methadone dose at or near the prior methadone dose increases the risk of overdose compared with a substantially lower dose. (n = 5)	0 (100)	6 (100)
Starting a tolerant client back on a methadone dose at or near the prior methadone dose is more satisfactory to the client compared with a substantially lower dose. (n = 6)	6 (100)	0 (0)
Starting a tolerant client back on a methadone dose at or near the prior methadone dose encourages better retention compared with a substantially lower dose. (n = 6)	6 (100)	0 (0)
Overall, I am satisfied with [restarting doses at or near last level] (n = 6)	6 (100)	0 (0)

© 2025 Christine PJ et al. *JAMA Network Open*.
