# Supplementary material for: Evaluation of a Novel Patient-Centered Methadone Restart Protocol
Source: JAMA Netw Open. 2025 Aug 28;8(8):e2529393. doi: 10.1001/jamanetworkopen.2025.29393 (PMC12395315; doi:10.1001/jamanetworkopen.2025.29393)
Supplement: Supplement 2. — Data Sharing Statement [file jamanetwopen-e2529393-s002.pdf]

Data Sharing Statement

Christine. Evaluation of a Novel Patient-Centered Methadone Restart Protocol. *JAMA Netw Open*. Published August 28, 2025. doi:10.1001/jamanetworkopen.2025.29393

Data
Data available: No

Additional Information
Explanation for why data not available: The data come from electronic health records with extensive PHI and cannot be shared outside of our institution.
